# Design, Synthesis and Antitumor Activity of Novel link-bridge and B-Ring Modified Combretastatin A-4 (CA-4) Analogues as Potent Antitubulin Agents

**DOI:** 10.1038/srep25387

**Published:** 2016-05-03

**Authors:** Yong-Tao Duan, Ruo-Jun Man, Dan-Jie Tang, Yong-Fang Yao, Xiang-Xiang Tao, Chen Yu, Xin-Yi Liang, Jigar A. Makawana, Mei-Juan Zou, Zhong-Chang Wang, Hai-Liang Zhu

**Affiliations:** 1 State Key Laboratory of Pharmaceutical Biotechnology, Nanjing University, Nanjing 210023, 163 Xianlin Road, P. R. China; 2 Guangxi University for Nationalities, Nanning 530006, People’s Republic of China; 3 Department of Pharmacology, School of Basic Medical Sciences, Nanjing Medical University, Nanjing 210029, China

## Abstract

A series of 12 novel acylhydrazone, chalcone and amide–bridged analogues of combretastatin A-4 were designed and synthesized toward tubulin. All these compounds were determined by elemental analysis, ^1^H NMR, and MS. Among them, compound **7** with acylhydrazone-bridge, bearing a benzyl at the indole-N position, was identified as a potent antiproliferative agent against a panel of cancer cell lines with IC_50_ values ranging from 0.08 to 35.6 μM. In contrast, its cytotoxic effects on three normal human cells were minimal. Cellular studies have revealed that the induction of apoptosis by compound **7** was associated with a collapse of mitochondrial membrane potential, accumulation of reactive oxygen species, alterations in the expression of some cell cycle-related proteins (Cyclin B1, Cdc25c, Cdc2, P21) and some apoptosis-related proteins (Bax, PARP, Bcl-2, Caspase3). The docking mode showed the binding posture of CA-4 and compound **7** are similar in the colchicine-binding pocket of tubulin, as confirmed by colchicine-tubulin competitive binding assay, tubulin polymerization inhibitory activity, extracellular protein expression determination assay and confocal immunofluorescence microscopy. *In vivo* study, compound **7** effectively inhibited A549 xenograft tumor growth without causing significant loss of body weight suggesting that compound **7** is a promising new antimitotic agent with clinical potential.

Microtubules are cytoskeletal structures that are formed by self-assembly of *α*- and *β*- tubulin heterodimers and involved in many cellular functions^1^,^2^. Their most important role is in the formation of the mitotic spindle which is essential to the mitotic division of cell. Consequently, microtubules have been important targets for the design and development of several potent natural and synthetic anticancer drugs such as paclitaxel, epothilone A, vinblastine, disorazol A1 and combretastatin A-4^2^,^3^,^4^,^5^,^6^,^7^ in the past decades.

CA-4, a natural product isolated by Pettit and coworkers in 1988 from the South African bush willow tree Combretum caffrum, strongly inhibits the polymerization of tubulin by binding to the colchicine-binding site^8^,^9^,^10^,^11^,^12^,^13^. Unlucky, poor solubility of CA-4 impinged its clinical development and required the preparation of more soluble derivatives such as CA-4P^14^. However, cardiovascular toxicity and neurotoxicity were dose limiting for CA-4P^15^. These significant side effects currently represent the main obstacles to broad clinical application of CA-4P. For this reason, it is important to develop other CA-4 structurally related compounds^16^,^17^,^18^. In this context, from a medicinal chemistry point of view, the structural simplicity of CA-4 offers a stimulating premise for the design of new and more potent compounds with improved pharmacological properties. So many research groups have been stimulated significant interest in a number of diverse ligands designed to mimic CA-4^19^,^20^,^21^,^22^,^23^,^24^,^25^(Fig. 1).

Based on the structure of CA-4 and its analogues, we divide CA-4 into three pharmacophore components, a linking bridge, A-ring containing 3,4,5-trimethoxy, and B-ring (Fig. 1). A-ring, with 3,4,5-trimethoxy moiety, plays an important role in inhibiting tubulin polymerization which is confirmed by the docking mode of CA-4 and tubulin (PDB ID: 1SA0) (Fig. 2).

This is the reason that considerable efforts had gone into modifying the link-bridge and B-ring. One example is the oxazoline (compound **e,** Fig. 1) that replacement of the ethenyl bridge and B-ring in CA-4 with pyrrole moiety and indole scaffold respectively. This prodrug showed potent antiproliferative activity. Also, Petti and coworkers reported that replacement of the carbon-carbon double bond in CA-4 could be replaced by carbonyl which resulted in a marked increase in water solubility and *in vivo* antitumor activity against murine solid tumors^26^; Gwaltney and coworkers show that the double bond bridge in CA-4 could also be replaced with a sulfonate to result in potent antitubulin agents^27^. Medarde and co-workers reported the synthesis and cytotoxic evaluation of indole-bridged combretastatin analogues^28^. Recently, Wang and coworkers in our lab have demonstrated that an indole group could replace the B ring to maintain potent cytotoxicity^29^.

In last these years, our research group has focused on the discovery and optimization of novel tubulin inhibitors as CA-4 analogues with high potency and acceptable pharmacologic and pharmacokinetic properties^30^,^31^,^32^. Especially, we were interested in the link-bridge whose modifications may be water-soluble to give improved physicochemical properties. Herein we wish to report the design and syntheses of acylhydrazone, chalcone and amide–bridged CA-4 derivatives with different alkyl-substituted indole-B-rings that showed potent antitumor activity *in vitro* and *in vivo*.

## Results

### Chemistry

The synthetic routes followed for the synthesis of the desired novel acylhydrazone, chalcone and amide-bridged CA-4 derivatives with different alkyl-substituted indole-B-rings are outlined in Fig. 3A–C, as elucidated in Section 3. All 12 synthesized compounds were reported and characterized for the first time by ^1^H NMR, elemental analysis, melting test, HPLC and mass spectroscopy, and results were in accordance with their depicted structures.

### Bioactivity

#### Compound 7 selectively inhibited cancer cell proliferation

All synthesized 12 derivatives were evaluated for their inhibitory effect of growth of six different human cancer cell lines (human liver carcinoma cell line HepG2, human epithelial cervical cancer cell line Hela, human breast carcinoma cell line MCF-7, carcinomic human alveolar basal epithelial cell line A549, human colorectal cancer cell line HT-29 and oral cancar SSC-4 cells), in comparison with the reference CA-4. As reported in Table 1, the antiproliferative activities of the tested compounds were less pronounced against HT-29 and SSC-4 cells than the other cell lines. Compound **7** with acylhydrazone-bridge, bearing a benzyl at the indole-N position exhibited the greatest antiproliferative activity among the tested compounds, with IC_50_ values of 0.08–35.6 μM, especially against A549 cells. On the whole, acylhydrazone–bridged compounds **4**–**7** showed more potent antiproliferative activities than compounds **10**–**13** and **16**–**19** with chalcone and amide–bridge, respectively. Interestingly, there was little difference observed in activity between compounds **10**–**13** and **16**–**19** against most cancer cell lines when compounds had the same groups at their indole-N. Notably, by comparison of compounds **4**–**6**, with the increase of the length of the alkyl chain, they presented gradually weak inhibition of cancer cell growth, suggesting that the stronger lipophilicity showed the weaker anticancer potency. And more, compounds **7**, **13** and **19** shared common substituted benzyl moieties, were from 5- to 100-fold more active than other compounds, implying that a bulky group on indole-N was well tolerated.

Compound 7 induced cell apoptosis through the regulation of the expression of apoptosis-related proteins along with mitochondrial dysfunction and intracellular ROS change in A549 cells and showed relative lack of toxicity toward normal cells.

To test whether cell apoptosis was involved in the inhibition of cell growth of A549, compound **7**-treated A549 cells were stained with Annexin V-FITC/PI staining and were analyzed by flow cytometry. As shown in Fig. 4A,B, compound **7** induced cell apoptosis in a dose-dependent and time-dependent manner. The percentage of apoptotic cell (Fig. 4A) significantly increased from 12.38% to 76.00% after treatment with different doses of 0.05, 0.1, 0.5 and 1.0 μM compound **7**. The result of Fig. 4B displayed that only 1.84% of the early apoptosis was induced in A549 cells treated with 0.8 μM compound **7** for 12 h. While, after continuing incubation, the total percentage of early and late apoptotic cells was 33.05% and 77.2% in the cases of 24 and 48 h, respectively. In contrast, only 0.01% of early and 0.07% late apoptotic cells were detected in the control cells. These results suggested that compound **7** could efficiently induce apoptosis of A549 cells in a dose and time-dependent.

The usefulness of a potential anticancer compound depends on not only its cytotoxicity in malignant cells, but also its relative lack of toxicity toward normal cells. We therefore evaluated the effects of compound **7** on three non-cancer cell line (human emborynic kidney 293T, human macrophage, and human fetal hepatocyte line L02) by the MTT assay. As shown in Fig. 4C, compound **7** almost had negligible effects on three normal cell lines below 135.5 μM.

Mitochondria play an essential role in the propagation of apoptosis. It is well established that at an early stage, apoptotic stimuli alter the mitochondrial transmembrane potential (Δψ_mt_) which can be monitored by the fluorescent dye 5,5′,6,6′-tetrachloro-1,1′,3,3′-tetraethyl-benzimidazolcarbocyanine (JC-1). JC-1 was capable of entering selectively into mitochondria and could reversibly change its color from green to red as the membrane potentials increase. As presented in Fig. 4D,E, compound **7** induced a time and concentration-dependent increase in the proportion of cells with depolarized mitochondria.

Mitochondrial membrane depolarization is associated with mitochondrial production of ROS. Therefore, we investigated whether ROS production changed after treatment with compound **7**. The results presented in Fig. 4F show that compound **7** induced the production of significant amounts of ROS in comparison with control cells and positive control, which agrees with the previously described dissipation of Δψ_mt_.

Bcl-2 and Bax protein expression was analyzed with western blot to learn the signal pathway that is induced by compound **7**. To elucidate the mechanism by which compound **7** exerts its apoptosis inducing effects on cells, A549 cells were treated with compound **7** at different concentrations (0.5, 1, and 2 μM) for 48 h. As shown in Fig. 4G, the level of Bax was efficiently up-regulated and the level of anti-apoptotic proteins (Bcl-2, Bcl-xl) was down-regulated. Caspases, which are proteolytic enzymes, are the central executioners of apoptosis, and their activation is mediated by various inducers. Synthesized as proenzymes, caspases are themselves activated by specific proteolytic cleavage reactions. Caspase-2, -8, -9, and -10 are termed apical caspases and are usually the first to be activated in the apoptotic process. Following their activation, they in turn activate effector caspases, in particular caspase-3. Following treatment of A549 cells with compound **7** for 48 h resulted in an up regulation of cleaved caspase-3 and PARP, which may be partially responsible for the apoptotic tendency of the A549 cells.

#### Compound 7 induced G2/M phase arrest and regulated the expression of G2/M-related proteins in A549 cells

It is well-known that tubulin-destabilizing agents block the cell cycle in the G2/M phase due to microtubule depolymerization and cytoskeleton disruption^33^. The excellent potency of compound **7** with respect to inhibit A549 cell proliferation prompted us to determine whether the cytotoxicity induced by compound **7** was due to cell cycle arrest, thus flow cytometry analysis of PI staining on cells treated by compound **7** was performed. A549 cells were treated with different concentrations (0, 0.05, 0.1 and 0.2 μM) compound **7** for 48 h and 0.12 μM compound **7** at different times (0, 12, 24, and 48 h), respectively. DNA content and cell cycle were determined using a FACS can laser flow cytometry a concentration of 0.05 μM (Fig. 5A), and the number of cells in G2/M fraction was significantly increased to 32.7%. When the concentration was increased to 0.1 and 0.2 μM, cells of 62.8% and 84.1% G2/M phase arrest were observed, respectively. Also, this similar phenomenon occurred after treatment with 0.2 μM compound **7** from 0 h to 48 h (Fig. 5B). These results indicated that compound **7** could induce cell-cycle progression arrest in G2/M phase in a dose- and time-dependent manner, which was in agreement with the activity of most antimitotic agents.

To further explore the underlying mechanisms of G2/M phase arrest induced by compound **7**, we examined the regulated effect on the expression of cyclins, Cdc2, Cdc25c and P21, which tightly control the cell cycle progression. Cell arrest at the prometaphase/metaphase to anaphase transition is normally regulated by the mitotic checkpoint. The major regulator of the G2 to M transition is M phase promoting factor (MPF), a complex made up of the catalytic subunit of cdc2 and the regulatory subunit of cyclin B1 which are held in an inactive state by phosphorylation of cdc2 at one negative regulatory sites (Tyr15)^34^. This and other regulatory complexes are activated at different points during the cell cycle and can also be regulated by several exogenous factors. As shown in Fig. 5C,D, cyclinB1 and cdc25c were down-regulated in a dose-dependent and time-dependent manner. Meanwhile, compound **7** increased p21 expression in a dose-dependent and time-dependent manner. Also, we observed there is a shift toward the cyclin-dependent kinase Cdc2 (Tyr15), which should result in inhibition of formation of the cdc2/cyclinB complex and increasing p21 expression.

#### Compound 7 bound to the colchicine-binding site of tubulin and inhibited tubulin polymerization

**Figure 1:**
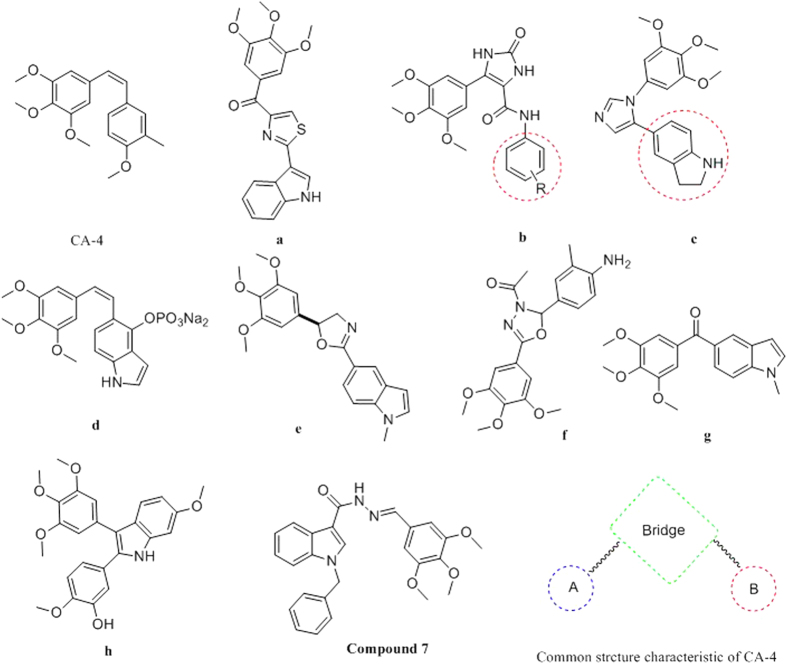
The chemical structure of CA-4 and its analogues.

Considering the critical role of microtubules in the cell cycle, tubulin has become an attractive target in anticancer drug discovery. To elucidate whether compound **7** targets the tubulin–microtubule system and how it binds, we docked compound **7** into the active center of tubulin (PDB code: 1SA0, http://www.rcsb.org/pdb/explore/explore.do?structureId = 1SA0) to investigate their binding mode. As shown in Fig. 6A,B, compound **7** is very similar to the pose of CA-4 (Fig. 1), with the 3,4,5,-trimethoxyphenyl rings placed in proximity to Cys 241 to form a hydrogen bond (angle O···H–O = 127.2°, distance = 2.62 Å). Furthermore, the acylhydrazone-bridge of compound **7** is placed toward the internal part of the pocket, and the hydrogen bond between this bridge and ASN 249 was does seem to establish specific interaction with tubulin. As expected, benzyl on the indole moiety is well-tolerated which interacted with the active center LYS 352 of *β*-tubulin by a π-cation bond. Also, the CDocker Energy (energy of the ligand-receptor complexes, -kcal/mol) of 13 compounds was also depicted in Fig. 6C, which agreed with the tubulin inhibitory trend and antiproliferative inhibitory trend (Table 1). To determine if the CA-4 analogues indeed bind to the colchicine-binding site of tubulin, we next performed a radioligand binding assay that confirmed dose-dependent inhibition of tubulin polymerization by competing with the [^3^H]colchicine binding to tubulin for compound **7**. The results showed that compound **7** strongly bound to the [^3^H]colchicine binding domain of tubulin compared with the known microtubule polymerization inhibitor CA-4 (IC_50_ = 4.96 μM), with IC_50_ value of 5.15 μM (Fig. 6D). The binding results are largely consistent with the molecular modeling predictions.

To test the inhibitory effect of compound **7** on microtubule assembly *in vitro*, the depolymerization was tested by the method originally described by Bonne *et al*. with some modifications^35^. In the control samples, which were not treated with any of the tested compounds, the fluorescence intensity at 340 nm increased with time. Incubation with either CA-4 or compound **7** resulted in various degrees of tubulin depolymerization (Fig. 6E). Compared to the control, compound **7** at 1.0 μM appeared to be slightly inhibiting tubulin polymerization. However, when concentration of compound **7** was increased to 5.0 μM, tubulin polymerization was nearly completely blocked just as 8.0 μM CA-4 did, suggesting that tubulin polymerization was inhibited by compound **7** in a concentration-dependent manner. The inhibitory mechanism of compound **7** on microtubule morphology was similar to that of CA-4 but not to paclitaxel. Next, to further investigate the effect of compound **7** on microtubule organization, we performed an *in vitro* microtubule assembly assay. As shown in Fig. 6F, compound **7** caused a decrease in the microtubule assembly (the curve shift to the left when compared with the control group), which was the same as CA-4 and in contrast to paclitaxel.

Considering the importance of the microtubule system in the maintenance of cellular morphology, an assay that involved the disruption of microtubule morphology was performed to reveal whether compound **7** could affect microtubule morphology in living cells. Figure 6G clearly showed that the cell membrane microtubule of colchicine (1.0 μM) -treated cells (positive control) showed depolymerization and solubilization. In contrast, the substantial stabilization of microtubules in paclitaxel (1.0 μM) -treated cells that were used as negative control in this assay. A549 cells were treated with vehicle exhibited a normal filamentous microtubule array, with microtubules extending from the central regions of the cell to the cell periphery. Compound **7** treated cells showed disorganized interphase microtubules, similar to CA-4. These morphological changes of microtubules indicate that compound **7** dramatically disrupts the microtubule morphology, which eventually leads to cell death.

#### Compound 7 effectively inhibited tumor growth *in vivo*

The *in vivo* antitumor efficacy of compound **7** was further investigated in a nude mouse xenograft model established with A549 cells. Mice were subcutaneously inoculated with injections of 5.0 × 10^6 ^cells/nude mice. After 12–14 days, tumor sizes were determined using micrometer calipers, and then nude mice with similar tumor volume (~100 mm^3^, eliminate mice with tumors that are too large or too small) were randomly divided them into four groups (with six nude mice/group). In two of the groups, compound **7** or reference CA-4P, dissolved in DMSO, was injected intraperitoneally at a dose of 30 mg/kg. Both drugs, as well as the vehicle control, were administered every other day for the entire period of observation.

The tumor volume measurement further confirmed the significant reduction in the compound **7** treatment group (P < 0.01). Compound **7** showed significant suppression of tumor growth reaching an average 47% (20 mg/kg) and 70% (30 mg/kg) reduction by the end of the observation period compared with administration of vehicle only, while treatment with CA-4P led to a 39% tumor reduction (Fig. 7A, the average weight of the excised tumors: the control group, 1.802 g; 20 mg/kg compound **7**-treated group, 0.951 g, 30 mg/kg compound **7**-treated group, 0.553 g, CA-4P-treated group, 1.102 g). Also, the tumor volume in CA-4P- or compound **7**-treated mice was less than that in negative control (saline) mice at the same measurement day (Fig. 7B). During the whole treatment period, no significant weight changes occurred in the treated animals (Fig. 7C), suggesting that compound **7** had minimal toxicity. All of these datas showed that compound **7** exhibits excellent antitumor ability in the inhibition of lung tumor growth in nude mice with A549 xenografts *via* the suppression of proliferation.

## Materials and Methods

### Chemistry section

(The detailed information is in Supplementary Information).

### Biological section

#### MTT assay^36^

The *in vitro* anticancer activities of the prepared compounds against HepG2, Hela, MCF-7, A549, HT-29 and SSC-4 cell lines were evaluated as described in the literature. Target tumor cells were grown to log phase in DMEM medium supplemented with 10% fetal bovine serum. After reaching a dilution of 1 × 10^5^ cells Ml^−1^ with the medium, 100 μL of the obtained cell suspension was added to each well of 96-well culture plates. Subsequently, incubation was performed at 37 °C in 5% CO_2_ atmosphere for 12 h before the cytotoxicity assessment. Tested samples at preset concentrations were added to 6 wells with CA-4 being employed as a positive reference. After 48 h exposure period, 25 μL of PBS containing 2.5 mg mL^−1^ of MTT (Sigma, USA) was added to each well. After 4 h, the medium was replaced by 150 μL DMSO to dissolve the purple formazan crystals produced. The absorbance at 570 nm of each well was measured with Synergy™ 2 Multi-Mode Microplate Reader. Cell viability (%) was calculated using the following equation: cell viability (%) = (Atreatment/Acontrol) × 100%. The data represented the mean of three independent experiments in triplicate and were expressed as means ± SD. The IC_50_ (the concentration that caused 50% inhibition of cell proliferations) was calculated by the Logit method.

#### Effects on tubulin polymerization^37^

Bovine brain tubulin was purified as described previously^37^. To evaluate the effect of the compounds on tubulin assembly *in vitro*, compounds of varying concentrations were preincubated with 10 μM tubulin in glutamate buffer at 30 °C and then cooled to 0 °C. After addition of GTP, the mixtures were transferred to 0 °C cuvettes in a recording BioTek Synergy 4 multifunction microplate spectrophotometer and warmed up to 30 °C and the assembly of tubulin was observed turbidimetrically (TU660, USA). The IC_50_ was defined as the compound concentration that inhibited the extent of assembly by 50% after 20 min incubation.

#### Detection of intracellular ROS

Intracellular ROS production was measured by use of active oxygen species assay kit (K0111, Beyotime, China). Pretreated with compound **7** for 48 h, A549 cells were collected and exposed to serum-free medium containing 10 mM DCFH-DA. After 30 min of incubation in the darkness, cells were washed with DMEM for three times, and then fluorescent intensity was measured by the flow cytometer with excitation and emission wavelengths of 488 and 525 nm, respectively.

#### Mitochondrial membrane potential (MMP) analysis

Alterations in MMP were analyzed by flow cytometry using the mitochondrial membrane potential assay kit with JC-1, which is a marker of mitochondrial activity (I0112, Beyotime, China). In normal undamaged nucleate cells, mitochondrion has a high MMP. Breakdown of MMP is often linked to early apoptosis. Cells containing J-aggregates have high MMP, and show red florescence (590 nm, FL-2 channel). Cells with low MMP are those in which JC-1 maintain smonomeric form, and show green florescence (530 nm, FL-1 channel). Briefly, after treatment with compound **7** for 48 h, A549 cells were collected and incubated with 0.5 ml JC-1 working solution for 20 min at 37 °C, then washed, resuspended in medium, and analyzed by flow cytometry. CCCP (carbonylcyanide-p-chlorophenolhydrazone) was used as positive control. Data were revealed as the monomers positive percentage of the treated cells to that of the untreated control.

### Apoptosis assay

To detect the apoptosis induced by compound **7**, A549 cells were seeded per well in 24-well plates and were incubated overnight. Then cells were treated with compound **7** at the four different concentrations (0.05 μM, 0.1 μM, 0.5 μM, and 1.0 μM separately) or treated cells with 0.8 μM compound **7** for 0, 12, 24, and 48 h. The percentages of apoptotic cells were estimated by staining with Annexin-V-FITC and with a PI Apoptosis Detection Kit (KGA105, Keygen biotech, China) according to the manufacturer’s instructions with some modifications. In brief, collected cells were washed once with PBS and subsequently washed once with binding buffer, and then stained with Annexin V-FITC and propidium iodide (PI) in the binding buffer for 20 min at room temperature in the dark. Apoptotic cells were quantified using a FACScancy to fluorometer (PT. MadagasiBrosa Inc. JI. BatangHari NO. 73, Propinsi Sumatera Utara, Indonesia) plotting at least 10,000 events per sample. To quantify the data, the frequencies in all quadrants were analyzed using flowjo 7.6.1 software. Both early apoptotic (Annexin V-positive, PI-negative) and late apoptotic (double positive of Annexin V and PI) cells were detected.

### Cell cycle analysis

A549 cells were seeded in 6-well plates (5.0 × 10^3 ^cells/well) and incubated at 37.8 °C for 12 h. Exponentially growing cells were then incubated with compound **7** at 0, 0.05, 0.1, and 0.2 μM. And in the time-dependent assays, exponentially growing cells were incubated with 0.18 μM compound **7** at 37.8 °C for 0, 12, 24, and 48 h. Cells were trypsinized, washed in PBS and centrifuged at 2000 rpm for 5 min. The pellet was then resuspended in 500 mL of staining solution (containing 5 mL Annexin V-PE and 5 mL PI in Binding Buffer from KGA511, Keygen biotech, China), mixed gently and incubated for 15 min at room temperature in dark. The samples were then read in a flow cytometer (Beckman Coulter, USA) at 488 nm excitation. Analyses were performed by the software supplied in the instrument.

#### Cell lysate extraction and western blot analyses^38^

Total cellular proteins are extracted with lysis buffer (50 mmol/l Tris [pH 7.4], 150 mmol/l NaCl, 1% Triton X-100, 1% deoxycholicphenyl methylsulfonyl fluoride, 1 mg/ml aprotinin, 5.0 mmol/l sodium pyrophosphate, 1.0 g/ml leupeptin, 0.1 mmol/l phenylmethylsulfonyl fluoride, and 1 mmol/l DTT). Protease inhibitors are added immediately before use. The cells are harvested at the end of treatment and extracted for western blot analysis as described previously. After washing twice with PBS, the cultured cells were collected and lysed in lysis buffer (100 mmol/l Tris-Cl, pH 6.8, 4% (m/v) SDS, 20% (v/v) glycerol, 200 mmol/l *β*-mercaptoethanol, 1 mmol/l phenylmethylsulfonyl fluoride, and 1 g/ml aprotinin). The lysates were centrifuged at 13,000 × g for 15 min at 4 °C. The concentration of total proteins was measured using the BCA assay method with Varioskan spectrofluorometer and spectrophotometer (Thermo) at 562 nm. Protein samples were separated with 15% SDS-PAGE and transferred onto the polyvinyldifluoride (PVDF) membranes (Millipore, IPVH 304 F0). Immune complexes were formed by incubation of proteins with primary antibodies overnight at 4 °C followed by IRDye^TM^ 800 conjugated second antibody for 1 hour at 37 °C. Immunoreactive protein bands were detected with an Odyssey Scanning System (LI-COR Biosciences, 9201-03). Antibodies: primary antibodies of cyclin B1, Cdc2, p-Cdc2 (Thr14/Tyr15), Cdc25C, and p21WAF1/Cip1 were all obtained from EPITOMICS (Abcam, Cambridge, UK); antibodies of Bax and Bcl-2 were obtained from Bioworld (Bioworld Technology, Inc., MN, USA); antibodies of caspase-3, and PARP were all obtained from Santa Cruz (Santa Cruz, CA); and antibody of GAPDH was from Boster (Wuhan, China). The secondary antibodies peroxidase-conjugated affinipure Goat Anti-Mouse IgG (H + L) (111-035-003), peroxidase-conjugated affinipure Goat Anti-Rabbit IgG (H + L) (111-035-003) were obtained from Jackson Immuno Research Laboratories, Inc. (USA).

#### Molecular Docking^39^

Molecular docking of compounds into the three dimensional X-ray structure of tubulin (PDB code: 1SA0) was carried out using the Discovery Studio (version 3.5) as implemented through the graphical user interface DS-CDOCKER protocol. The 3D structure of 1SA0 in docking study was downloaded from Protein Data Bank. The three-dimensional structures of the aforementioned compounds were constructed using Chem. 3D ultra 12.0 software [Chemical Structure Drawing Standard; Cambridge Soft corporation, USA (2010)], then they were energetically minimized by using MMFF94 with 5000 iterations and minimum RMS gradient of 0.10. For protein preparation, the hydrogen atoms were added, and the water and impurities were removed. The whole tubulin was defined as a receptor and the site sphere was selected based on the ligand binding location of colchicine, then the colchicine molecule was removed and 3D structure of compounds was placed during the molecular docking procedure. Types of interactions of the docked protein with ligand were analyzed after the end of molecular docking. Each compound would retain 10 poses, and were ranked and selected by CDOCKER_INTERACTION_ENERGY.

#### [^3^H] Colchicine–tubulin binding assay^38^

One micromolar radiolabeled colchicine [ring C, Methoxy-^3^H] (Perkin-Elmer), 1% DMSO and various concentrations of test compounds in 50 μL G-PEM buffer containing 80 mmol/L PIPES (pH 6.8), 1 mmol/L EGTA, 1 mmol/L MgCl_2_ and 1 mmol/L GTP, and 5% glycerol were incubated with 1 mmol/L tubulin (>99% pure,; Cytoskeleton, Inc.; 0.2 mg/mL) at 37 °C for 60 min. The binding solutions were filtered through a stack of 2 DEAE-cellulose filters and washed twice. The radioactivity in the filtrates was determined by liquid scintillation spectrometry (Perkin-Elmer Wallac). Nonlinear regression was used to analyze the data using GraphPad Prism.

#### Tubulin polymerization assay^40^

Tubulin polymerization was determined with the tubulin polymerization assay kit (Cytoskeleton, Denver, CO which was purchased from Cytoskeleton, Beyotime, China. Tubulin isolated from porcine brain tissue was used in this commercial kit.) It is based on the principal that light is scattered by microtubules to an extent that is proportional to the concentration of the microtubule polymer. Compounds that interact with tubulin will alter the polymerization of tubulin, and this can be detected using a spectrophotometer. Tubulin polymerization dynamics was monitored through measuring the change of absorbance at 340 nm every 2 min for 30 min at 37 °C on a spectrophotometer (Perkin-Elmer).

#### Flow cytometry analysis of the expression of extracellular polymerized tubulin^7^

A549 cells were seeded in 6-well plates and treated with 1 μM compound **7**, 1 μM colchicine and 1 μM paclitaxel for 48 h. Then, cells were harvested, washed once with PBS and blocked with 1% BSA for 30 min on ice, then were centrifuged (2800 rpm at 4 °C for 5 minutes) to remove supernatant. After that, cells were incubated with *β*-tubulin antibody (AT819, Beyotime, China) for 1 h on ice and were centrifuged (2800 rpm at 4 °C for 5 minutes) to remove supernatant. Next, cells were incubated with CY5.5-linked anti-rabbit IgG (A7016, Beyotime, China) on ice for 1 h and were also centrifuged (2800 rpm at 4 °C for 5 minutes) to remove supernatant. Finally, cells were then resuspended in PBS and the expression level of polymerized tubulin was analysed by flow cytometry (BD, USA).

#### Confocal microscopy assay^41^

A549 cells were incubated on cover slips in the 6-well plates to 70% confluence and treated with 1 μM compound **7**, 1 μM CA-4, 1 μM paclitaxel and 1 μM colchicine for 48 h, respectively. After incubating for 48 h, A549 cells were washed with PBS for three times and fixed with 4% paraformaldehyde for 20 min, permeabilized with cold methanol for another 10 min. After that, the cells were blocked with 5% BSA for 1 h. Subsequently, the cells were washed with PBS, and incubated with anti-tubulin antibody in 3% BSA (1:200 Cytoskeleton, Inc.) overnight at 4 °C. After being washed with PBS for three times, each cover slip was added 200 μL of Cy3-labeled goat anti-mouse IgG (H + L) in 3% BSA (1:500, Cytoskeleton, Inc.) and incubated for 1 h at room temperature. At last, A549 cells was stained with 200 μL of DAPI for 5 min and observed under an confocal microscope (Olympus, USA).

#### Evaluation of *in vivo* anti-tumor activity^42^

This experiment was conducted in accordance with the guideline issued by the State Food and Drug Administration (SFDA of China). The animals were housed and cared for in accordance with the guidelines established by the National Science Council of Republic China. All experimental protocols were approved by Animal Care and Use Committee of Nanjing University.

Male BALB/c nude mice, five weeks old and weighing 18–22 g, were supplied by Shanghai Laboratory Animal Limited Company. The mice were raised in air-conditioned rooms under controlled lighting (12 h light-dark cycle at 24 °C) and were fed with standard laboratory food and water ad libitum. Before injection into the mice, the A549 cells were harvested by trypsinization and washed three times with cold serum-free medium and then injected in a total volume of 0.1 mL using a 1 mL latex-free syringe (BD) within 30 min of harvest. Mice were inoculated subcutaneously with A549 cells (5.0 × 10^6^) on their shoulders. When the tumor had increased to 100 mm^3^, the mice were equally randomized into 4 groups (with 6 mice/group): saline tumor control group; compound **7** 15 mg/kg/2 days group; compound **7** 30 mg/kg/2 days group; and CA-4P 15 mg/kg/2 days positive control group. The control group received 0.9% normal saline. Tumor size was measured once every 2 days in two per-pendicular dimensions with Vernier calipers and converted to tumor volume (TV) using the formula: (ab^2^)/2, where *a* and *b* refer to the longer and shorter dimensions, respectively. The body weight of the animals was measured twice a week to assess toxicity of the treatments at the same time as the tumor dimension measurement and the mortality was monitored daily. After the treatments for eight weeks, the animals were euthanized by cervical dislocation. This study conformed to the Guide for the Care and Use of Laboratory Animals as published by the US National Institutes of Health and was approved by the Institutional Ethics Review Board of Nanjing University.

### Statistical analysis

Statistical analysis was performed with Origin Version 9.0 statistic software package. Data were expressed as means ± standard deviation (SD). Comparisons between groups were performed with analysis of non-parametric test. A value of P < 0.05 was considered statistically significant.

## Discussion

Microtubules are cytoskeletal filaments consisting of *α*-, *β*- tubulin heterodimers and are involved in a wide range of cellular processes such as cell shape organization, transportation of vesicles, mitochondria, and other cellular organs, cell signaling, cell division, and mitosis, which are required for cell life cycle^43^,^44^. Tubulin, the major structural component of microtubules, is a target for the development of anticancer agents. CA-4 can bind to the colchicine binding site of tubulin to block microtubule assembly, causing rapid vascular shutdown and cell death in the tumor^45^. The water-soluble phosphate prodrug form (CA-4P, also known as fosbretabulin) is in phase II/III clinical trials either alone or in combination with traditional chemotherapeutic agents or with radiotherapy^46^. In our present study, we aimed at whether potent anticancer activity of compounds was related to the intracellular microtubules depolymerization and reconstitutation.

Except CA-4P, despite the intense interest and the large number of potent derivatives of CA-4 that have been discovered that aimed at targeting the colchicine-binding site of tubulin, none of these inhibitors has reached the clinical stage. Thus, challenges remain in developing CA-4 analogues with improved pharmacological properties for eventual acceptance in the clinic.

**Figure 3:**
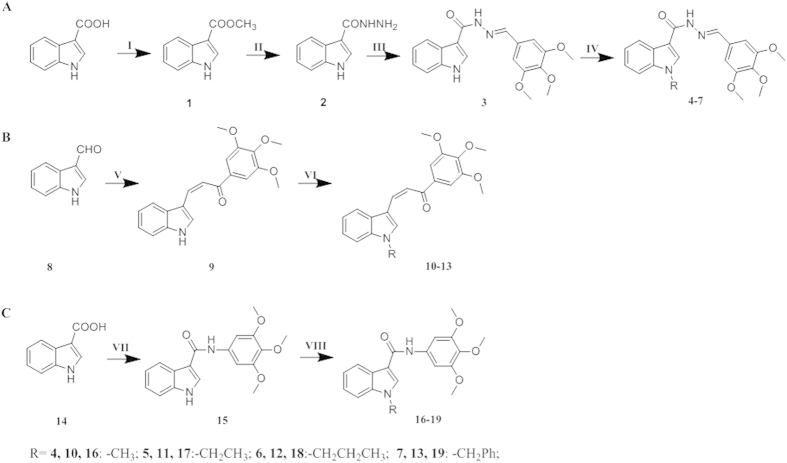
(**A**) Synthetic route for compounds **4**–**7**. (I) methanol, concentrated sulfuric acid, 65 °C, 12 h; (II) hydrazine hydrate (85%), ethanol, 80 °C, 4–5 h; (III) ethanol, acetic acid, 80 °C, 4–5 h; (IV) NaH, different haloalkanes, THF, 0 °C to room temperature. (**B**) Synthetic route for compounds **10–13**. (I) EtOH, 40% NaOH, 0 °C, 30 min; room temperature, 4 h; (II) NaH, different haloalkanes, THF, 0 °C to room temperature. (**C**)Synthetic route for compounds **16–19**. (VII) EDC∙HCl, HOBt, rt, 8–10 h. (VIII) NaH, different haloalkanes, THF, 0 °C to room temperature.

**Figure 4:**
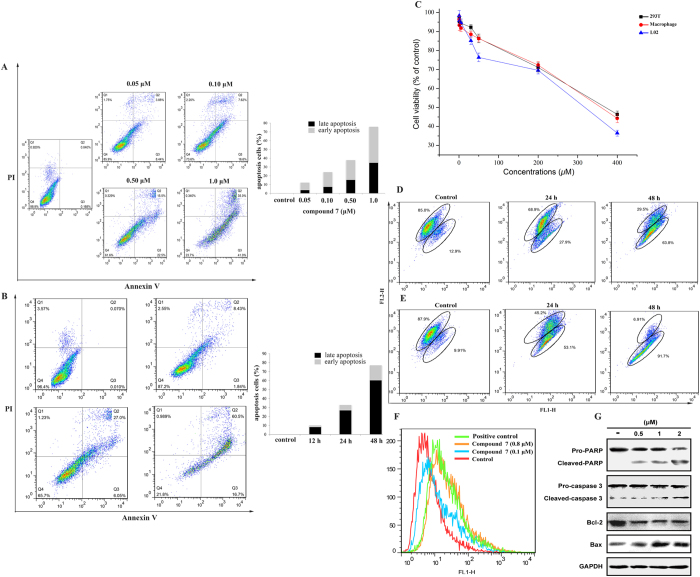
(**A**) A549 cells treated with 0.05, 0.1, 0.5, 1.0 μM compound **7** for 48 h were collected and processed for analysis. (**B**) A549 cells treated with 0.8 μM compound **7** for different times (0, 12, 24, and 48 h) were collected and analyzed. (**C**) Cytotoxicity of compound **7** toward three non-cancer cell lines (293T, Macrophage, and L02) for 48 h. Images are representative of three independent experiments. Data are mean ± SD of three independent experiments. (**D**) Effect of 0.5 μM compound **7** on the MMP decrease in A549 cell for 24 h and 48 h. (**E**) Effect of 1.0 μM compound **7** on the MMP decrease in A549 cell for 24 h and 48 h. (**F**) The levels of intracellular ROS in A549 cells were measured by flow cytometry. (**G**) Western blot analysis of the levels of the apoptosis-related proteins Bax, PARP, Bcl-2, and Caspase3 after A549 cells were treated with or without compound **7** at 0.5 μM, 1 μM, and 2 μM for 48 h.

**Figure 5:**
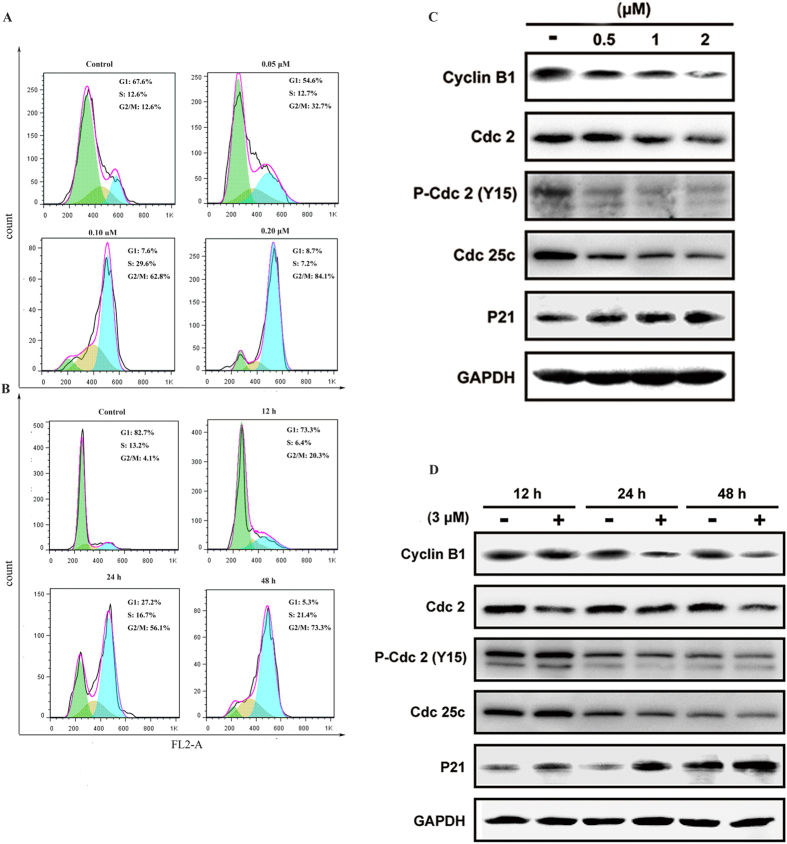
Compound 7 induces G2/M phase arrest and regulates the G2/M-related protein expression in A549 cells. (**A**) Cells treated with 0, 0.01, 0.1, and 0.2 μM compound **7** for 48 h were collected and processed for analysis. (**B**) Cells treated with 0.18 μM compound **7** for different times (0, 12, 24, and 48 h) were collected and analyzed. The percentage and images of cell cycle distribution in G2 phase were shown as representative of three independent experiments. (**C**) Western blot analysis of the levels of the G2/M-related proteins Cyclin B1, Cdc25c, Cdc2, and P21. A549 cells were treated with or without compound **7** at 0.5 μM, 1.0 μM, and 2.0 μM for 48 h. (**D**) Western blot analysis of the levels of the G2/M-related proteins Cyclin B1, Cdc25c, Cdc2, and P21. A549 cells were treated with or without 3.0 μM compound **7** for 12 h, 24 h and 48 h.

**Figure 6:**
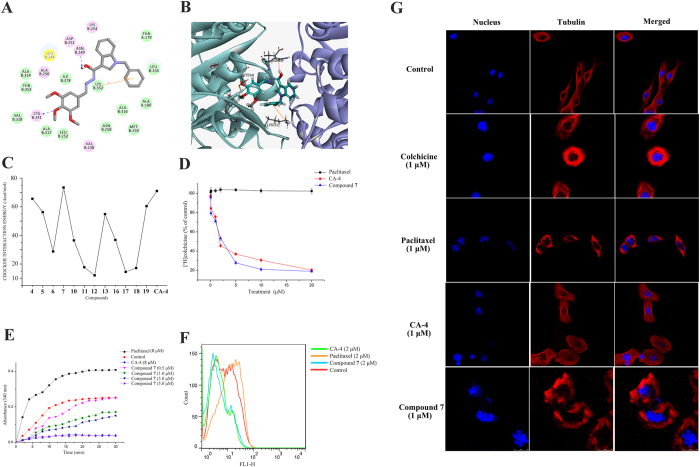
Compound 7 binds to the colchicine-binding site of tubulin and inhibits microtubule polymerization. (**A**) 2D model of the interaction between compound **7** and tubulin. (**B**) Binding mode of 3D model of the interaction between compound **7** and tubulin. (**C**) The CDOCKER INTERACTION ENERGY (-kcal/mol) obtained from the docking study of all compounds by the CDOCKER protocol (Discovery Studio 3.5, Accelrys, Co. Ltd). (**D**) Effect of compound **7** on tubulin binding of [^3^H] colchicine. Tubulin (>99% pure) was incubated with tritiated tubulin binders in the presence of different concentrations compound **7**, CA-4 or paclitaxel. (**E**) Effects of compounds **7** on microtubule morphology. Polymerization of tubulin at 37 °C in the presence of compound **7** (0.5, 1.0, 3.0, 5.0 μM), CA-4 (8.0 μM) and paclitaxel (8.0 μM). (**F**) Compound **7** affected microtubule assembly *in vitro*. After 48 h treatment with compound **7**, paclitaxel and CA-4, polymerized tubulin on the cell membrane was immunoblotted with *β*-tubulin antibody and detected by flow cytometry. (**G**) Effects of compound **7** (1.0 μM), paclitaxel (1.0 μM), CA-4 (1.0 μM) and colchicine (1.0 μM) on interphase microtubules of A549 cells. Microtubules tagged with rhodamine (red) and nuclei tagged with DAPI (blue) were observed under a confocal microscope. The photomicrographs shown are representative of at least three independent experiments performed.

**Figure 7:**
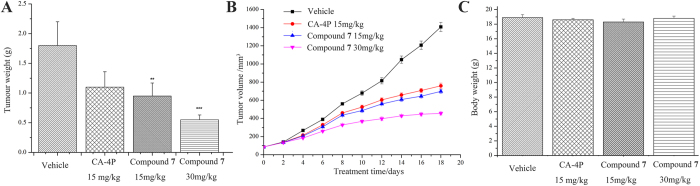
Compound 7 has a potential antitumor effect and low toxicity A549 xenograft tumors. (**A**) The tumor volumes in mice treated with saline, CA-4P, compound **7** (15 mg/kg) and compound **7** (30 mg/kg). (**B**) The tumor weight in mice treated with saline, CA-4P, compound **7** (15 mg/kg) and compound **7** (30 mg/kg). (**C**) The nude mice weight was examined every other day. Data are presented as the mean ± SD. *P < 0.05, **P < 0.01, ***P < 0.001.

In this investigation, according to the Fig. 3A–C a series of 12 novel CA-4 analogues were designed and synthesized that exerted a broad spectrum of anti-proliferative effects against HepG2, Hela, MCF-7, A549, HT-29 and SSC-4 *in vitro*. The observed antiproliferative activities depended on the link-bridge between 3,4,5-trimethoxyphenyl-A ring and indole-B ring. Also, the benzyl group at the indole-N enhanced biological activity. Compound **7** modified from CA-4, with acylhydrazone–bridge, bearing a benzyl at the indole-N position, presented satisfied antiproliferative potency. In contrast, at concentrations below 140 μM, compound **7** showed lower cytotoxicity against three normal cells.

Presently, there is one established mechanism that is known to communicate between mitotic arrest and apoptosis^47^, in which Bcl-2 is a key factor. As with other microtubule destabilizing agents, the levels of the pro-apoptotic proteins Bax was strongly up-regulated after the treatment of compound **7**, whereas the expression of the anti-apoptotic proteins Bcl-2 was dramatically down-regulated. Subsequently, we observed that the caspase-3 and PARP were cleaved. Moreover, compound **7** induces apoptosis that is dependent primarily on mitochondrial dysfunction and ROS damage, which are embodied in the MMP collapse and intracellular ROS accumulation. Altogether, these results indicate that these compounds induced apoptosis through the mitochondrial pathway.

Just like most microtubule-interacting agents, compound **7** induced cell cycle arrest at the G2/M phase accompanied with a decrease in mitotic cells and an increase in apoptotic cells and led to cell apoptosis. The progression of cell from G2 to M phase requires the coordination of several key regulatory proteins including cyclinB1 and Cdc2. Phosphorylation of Cdc25 directly stimulates its phosphatase activity, which is necessary to activate the Cdc2/cyclin B1 complex and for entry into mitosis. Compound **7** treatment leads to a decrease in the protein levels of cyclin B1 with a concomitant decrease in cdc2 and cdc25c activity. Compound 7 also induces the p21 expression. The peformance in these proteins is consistent with cell cycle arrest in mitosis.

Molecular modeling analysis indicates compound **7** binds to the colchicine site of tubulin, showing greater binding affinity than CA-4. Competitive binding assay confirms that the acylhydrazone -biriged analogues bind to the colchicine site with affinities similar to CA-4. Also, *in vitro* tubulin polymerisation assay, immunofluorescence studies in cells and expression of extracellular polymerized tubulin assay all demonstrated compound **7** interacted with tubulin at the colchicine active center, causing disruption of tubulin polymerization, and interferes with the normal formation of mitotic spindles *via* the depolymerization of microtubules.

Importantly, the antitumor efficacy of compound **7** was demonstrated in the A549 xenograft tumor model. Its satisfactory bioavailability, which might mostly due to excellent solubility in water of compound **7** with the strong polarity acylhydrazone–bridge moiety, contributes to its highly effective anti-tumor activity *in vivo*.

In summary, this is the first report that compound **7** of CA-4 analogues containing acylhydrazone–bridge, bearing a benzyl at the indole-N position, could inhibit tubulin polymerization with potent antitumor activity. The findings also provide the molecular basis for compound **7** to act as an antitumor for human carcinoma in the future and promote us to make further preclinical evaluation as a potential chemotherapeutic agent.

## Additional Information

**How to cite this article**: Duan, Y.-T. *et al*. Design, Synthesis and Antitumor Activity of Novel link-bridge and B-Ring Modified Combretastatin A-4 (CA-4) Analogues as Potent Antitubulin Agents. *Sci. Rep*. **6**, 25387; doi: 10.1038/srep25387 (2016).

## Supplementary Material

Supplementary Information

## Figures and Tables

**Figure 2:**
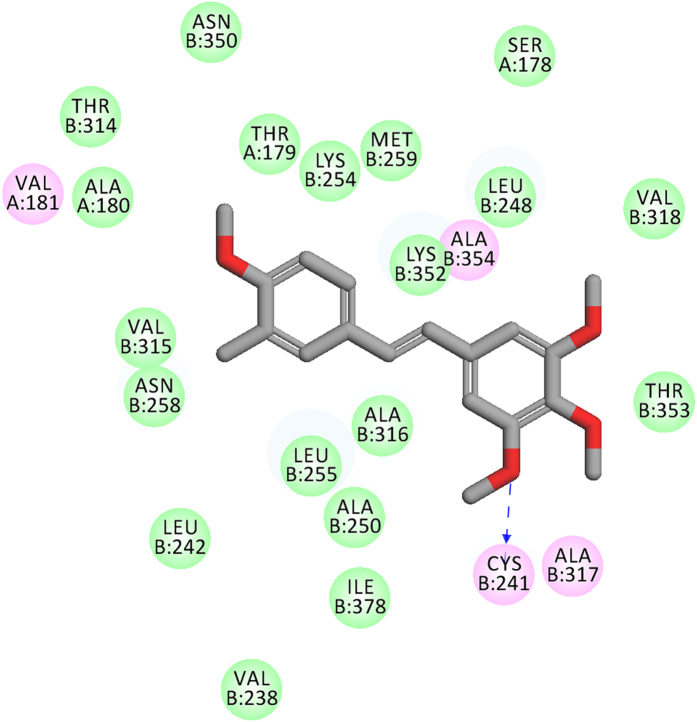
Binding mode of CA-4 and colchicine-site of tubulin (PDB ID: 1SA0).

**Table 1:** IC_50_ Values (*μ*M (SD^a^) of synthesized compounds and CA-4.

Cmp	Cell type (IC_50_ *μ*M (SD^a^)						
	HepG2	Hela	MCF-7	A549	HT-29	SSC-4	Tubulin inhibition
**4**	2.43 ± 0.23	8.45 ± 1.87	3.21 ± 0.76	0.29 ± 0.04	26.7 ± 1.89	43.2 ± 3.98	14.78
**5**	10.64 ± 1.98	23.76 ± 2.23	14.21 ± 1.11	1.18 ± 0.11	56.78 ± 2.10	55.23 ± 3.48	23.43
**6**	38.98 ± 3.23	56.89 ± 4.89	28.90 ± 3.23	9.89 ± 1.16	69.98 ± 3.76	79.90 ± 2.78	55.67
**7**	0.63 ± 0.12	5.89 ± 1.40	0.64 ± 0.11	0.08 ± 0.01	14.2 ± 0.04	35.6 ± 1.91	4.46
**10**	19.6 ± 1.98	23.87 ± 5.89	29.86 ± 3.21	12.1 ± 1.2	44.52 ± 2.87	43.2 ± 3.3	49.88
**11**	27.8 ± 2.23	34.23 ± 1.19	45.45 ± 5.67	27.9 ± 6.71	49.89 ± 6.98	57.81 ± 4.67	124.76
**12**	53.89 ± 2.81	67.9 ± 4.47	69.8 ± 5.43	37.9 ± 3.61	78.9 ± 3.51	89.98 ± 9.98	>200
**13**	2.98 ± 0.34	4.78 ± 1.43	4.54 ± 0.96	2.78 ± 0.92	34.67 ± 5.34	25.68 ± 2.78	24.77
**16**	17.5 ± 1.46	26.84 ± 6.81	27.45 ± 2.41	17.6 ± 1.9	46.42 ± 2.83	45.5 ± 2.6	46.78
**17**	28.3 ± 3.33	35.7 ± 2.34	48.3 ± 6.58	26.47 ± 6.2	39.4 ± 3.45	47.2 ± 4.33	>200
**18**	62.8 ± 3.65	77.4 ± 5.78	59.8 ± 3.43	40.2 ± 4.41	82.2 ± 3.7	90.3 ± 6.76	>200
**19**	3.68 ± 0.36	5.74 ± 1.06	3.61 ± 0.46	2.69 ± 0.40	32.68 ± 3.31	29.48 ± 3.42	19.89
**CA-4**	0.46 ± 0.07	5.52 ± 0.82	0.81 ± 0.11	0.13 ± 0.02	15.6 ± 0.06	27.2 ± 1.78	9.76

^a^SD: standard deviation. All experiments were independently performed at least three times.
