# Supplementary material for: Design, Synthesis and Antitumor Activity of Novel link-bridge and B-Ring Modified Combretastatin A-4 (CA-4) Analogues as Potent Antitubulin Agents
Source: Sci Rep. 2016 May 3;6:25387. doi: 10.1038/srep25387 (PMC4853715; doi:10.1038/srep25387)

Design, Synthesis and Antitumor Activity of Novel link-bridge and B-Ring Modified Combretastatin A-4 (CA-4) Analogues as Potent Antitubulin Agents

Yong-Tao Duan[†1], Ruo-Jun Man[†1], Dan-Jie Tang[†1], Yong-Fang Yao[1], Xiang-Xiang Tao[1], Chen Yu[1], Xin-Yi Liang[1], Jigar A. Makawana[1], Mei-Juan Zou*[2], Zhong-Chang Wang*[1], Hai-Liang Zhu*[1,2]

General procedure for preparation of compounds **4-7**

Concentrated sulfuric acid (1.2 mL) was added slowly with vigorous agitation to a mixture of 20 mmol Indole-3-carboxylic acid and 32 mL of methanol. The mixture was then refluxed for 12 h. Excess methanol was removed by evaporation and the residue was washed with water for three times. The product was dried over anhydrous $MgSO_4$, collected by filtration, and distilled. Equimolar quantities (15 mmol) compound **1** and the 85% hydrazine monohydrate were dissolved in anhydrous ethanol (25 mL) and vigorously stirred at 80 ˚C under oil bath for 4-5 h. The crude was precipitated from the solvent, collected using suction filtration, dried, and recrystallization in ethanol. Then to a stirred solution of compound **2** (2.0 mmol) and 3,4,5-trimethoxybenzaldehyde (2.0 mmol) in ethanol (30.0 mL), water (2 mL) was added followed by drop-wise addition of glacial acetic acid (0.4 mL). Then, the resulting mixture was stirred at room temperature until the target product precipitated from the solvent, collected using suction filtration and dried, and followed by recrystallization in ethanol. NaH (60% dispersion in mineral oil, 2.0 mmol) was added in portions to a stirred solution of compound **7** (2.0 mmol) in anhydrous THF (10.0 mL) cooled in an ice bath. The resulting mixture was then allowed slowly to warm to r.t. After stirring for 30 min, different haloalkanes (3.0 mmol) in anhydrous THF (3.0 mL) was added drop wise. When TLC monitoring showed complete consumption of the starting material, the reaction mixture was evaporated under reduced pressure to leave a residue that was treated with ice water (100 mL). The resulting solid was filtrated off and recrystallized from acetone/petroleum ether (60-90 ˚C) to give the desired compounds **4-7**.

Methyl-N'-(3,4,5-trimethoxybenzylidene)-1*H*-indole-3-carbohydrazide

Yellow solid, yield 78%, m.p. 164~166˚C; ^{1}H NMR (DMSO-d_6, 300 MHZ) δ: 11.42 (s, 1H, NH), 8.24-8.16 (m, 3H, ArH, CH), 7.55 (d, *J* = 6.1HZ, 1H, ArH), 7.27 (t, *J* = 5.8 HZ, 1H, ArH), 7.21-7.18 (m, 1H, ArH), 7.03 (s, 2H, ArH, CH), 3.89 (d, *J* =7.8 HZ, 9H, CH_3), 3.72(s, 3H, CH_3). ESI-MS: m/z 367.40 (M^+). Anal.Calcd for $C_{20}H_{21}N_3O_4$: C, 65.38; H, 5.76; N, 11.44; Purity: 96%.

1-Ethyl-N'-(3,4,5-trimethoxybenzylidene)-1*H*-indole-3-carbohydrazide

Yellow solid, yield 67%, m.p. 162~163˚C; ^{1}H NMR (DMSO-d_6, 300 MHZ) δ: 11.41(s, 1H, NH),8.24 (s, 2H, ArH, CH),7.59 (d, *J* =6.2 HZ, 2H, ArH), 7.27~ 7.18 (m, 2H, ArH), 7.03 (s, 2H, ArH, CH), 4.34~ 4.28 (m, 2H, CH_2), 3.86 (s, 6H, CH_3),3.72 (s，3H, CH_3), 1.45 (t, *J* =5.5 HZ, 3H, CH_3).ESI-MS: m/z 381.43 (M^+). Anal.Calcd for $C_{21}H_{23}N_3O_4$: C, 66.13; H, 6.08; N, 11.02. Purity: 98%.

1-Propyl-N'-(2,4,6-trimethoxybenzylidene)-1*H*-indole-3-carbohydrazide

Red solid, yield 79%, m.p. 161~162˚C; ^{1}H NMR (DMSO-d_6, 300 MHZ) δ: 11.40(s, 1H, NH), 8.24 (s, 2H, ArH, CH), 7.26 (t, *J* = 5.8 HZ, 1H, ArH),7.19 (t, *J* = 5.7HZ, 2H, ArH, CH), 7.03 (s, 2H, ArH), 4.25-4.23 (m, 2H, CH_2), 3.87 (s, 6H, CH_3), 3.72 (s, 3H, CH_3), 1.89~1.80 (m, 2H, CH_2), 0.90 (t, *J* =5.4 HZ, 3H, CH_3). ESI-MS: m/z 395.45 (M^+). Anal.Calcd for $C_{22}H_{25}N_3O_4$: C, 66.82; H, 6.37; N, 10.63; Purity: 97%.

1-Benzyl-N'-(3,4,5-trimethoxybenzylidene)-1*H*-indole-3-carbohydrazide

Yellow solid, yield 70%, m.p. 154~155˚C; ^{1}H NMR (DMSO-d_6, 300 MHZ) δ: 11.50 (s, 1H, NH), 8.68(s, 2H, ArH),8.25(2, 1H, ArH, CH),7.56 (d, *J* = 5.1 HZ, 2H, ArH), 7.35 (d, *J* = 6.6 HZ, 2H, ArH, CH), 7.23(s, 3H, ArH), 7.01 (s, 2H, ArH), 5.57 (s, 2H, CH_2), 3.83 (d, *J* =9.6 HZ, 6H, CH_3), 3.72 (d, *J* = 9.4 HZ, 3H, CH_3). ESI-MS: m/z 398.41 (M^+). Anal.Calcd for $C_{23}H_{18}N_4O_3$: C, 69.34; H, 4.55; N, 14.06; Purity: 98%.

General procedure for preparation of compounds **10-13**

Indole-3-carbaldehyde (10 mmol) and 3',4',5'-trimethoxyacetophenone (10 mmol) in ethanol (25 mL) were mixed gently at 0 ˚C. Then 40% NaOH (5 mL) was added and stirred for 30 min. The mixture was placed to room temperature to continue the reaction for 4 h and the solid was filtered, washed with water and dried to obtain compound **9**. The step II is just similar to step IV above mentioned.

(*E*)-3-(1-methyl-1*H*-indol-3-*yl*)-1-(3,4,5-trimethoxyphenyl)prop-2-en-1-one

Yellow solid, yield 87%, m.p. 160~162°C; ^{1}H NMR (DMSO-d_6, 300 MHZ) δ: 8.18 (S, 1H, ArH), 8.09~8.01 (m, 5H, ArH), 7.65~7.55 (m, 2H, ArH, CH), 7.38~7.25 (m, 4H, ArH, CH), 3.93~3.87 (m, 9H, CH_3), 3.77~372 (m, 3H, CH_3), ESI-MS: m/z 351.12 (M^+). Anal.Calcd for $C_{21}H_{11}NO_4$: C, 71.78; H, 6.09; N, 3.96. Purity: 97%.

(*E*)-3-(1-ethyl-1*H*-indol-3-*yl*)-1-(3,4,5-trimethoxyphenyl)prop-2-en-1-one

Yellow solid, yield 73%, m.p. 155~158°C; ^{1}H NMR (DMSO-*d*6, 300 MHZ) δ: 8.23 (S, 1H, ArH), 8.09~8.01 (m, 2H, ArH), 7.65~7.61 (m, 2H, ArH), 7.38~7.25 (m, 3H, ArH, CH), 4.32~4.26 (m, 2H, CH_2), 3.92~3.86 (m, 6H, CH_3), 3.78~3.72 (m, 3H, CH_3), 1.45 (t, J = 4.52 HZ, 3H,CH_3), ESI-MS: m/z 275.13 (M^+). Anal.Calcd for $C_{22}H_{23}NO_4$: C, 72.32; H, 6.32; N, 3.83. Purity: 96%.

(*E*)-3-(1-propyl-1*H*-indol-3-*yl*)-1-(3,4,5-trimethoxyphenyl)prop-2-en-1-one

Light yellow solid, yield 85%, m.p. 162~164°C; ^{1}H NMR (DMSO-*d*6, 300 MHZ) δ: 8.23 (S, 1H, ArH), 8.09~8.01 (m, 2H, ArH), 7.66~7.62 (m, 2H, ArH, CH), 7.38~7.23 (m, 4H, ArH , CH), 4.23~4.20 (m, 2H, CH_2), 3.92~3.86 (m, 6H, CH_3), 3.78~3.72 (m, 3H, CH_3), 1.87~1.81 (m, 3H, CH_2), 0.87 (t, 3H, J = 4.50 HZ, CH_3), ESI-MS: m/z 337.15 (M^+). Anal.Calcd for $C_{23}H_{25}NO_4$: C,72.80; H, 6.64; N, 3.65. Purity: 97%.

(*E*)-3-(1-benzyl-1*H*-indol-3-*yl*)-1-(3,4,5-trimethoxyphenyl)prop-2-en-1-one

Red solid, yield 85%, m.p. 160~163°C; ^{1}H NMR (DMSO-*d*6, 300 MHZ) δ: 8.38 (s, 1H, ArH), 8.11~8.03 (m, 2H, ArH), 7.70~7.58 (m, 2H, ArH), 7.39~7.24 (m, 11H, ArH, CH), 5.50 (S, 2H, CH_2), 3.92~3.86 (m, 6H, CH_3), 3.78~3.73 (m, 3H, CH_3), ESI-MS: m/z 337.15 (M^+). Anal.Calcd for $C_{27}H_{25}NO_4$: C, 75.86; H, 5.88; N, 3.28. Purity:95%.

General procedure for preparation of compounds **16-19**

An equimolar (1.0 mol) indole-3-carboxylic acid and 3,4,5-trimethoxyaniline in anhydrous CH_2Cl_2 was stirred for 8 h with carbodiimide hydrochloride (1.5 mmol) and N-hydroxybenzotriazole (0.5 mmol). Then reaction mixture was extracted twice using ethyl acetate and water. The organic layer was evaporated and recrystallized from ethanol to give the title compound **15**. The step II is also similar to step IV.

1-Methyl-N-(3,4,5-trimethoxyphenyl)-1*H*-indole-3-carboxamide

Light yellow solid, yield 87%, m.p. 134~136°C; ^{1}H NMR (DMSO-*d*6, 300 MHZ) δ:

9.03 (S, 1H, ArH), 8.44~8.21 (m, 1H, ArH), 7.58~7.50 (m, 1H, ArH), 7.38~7.32 (m, 1H, ArH), 7.36~7.24 (m, 2H, ArH, CH), 7.22~7.19 (m, 1H, ArH), 6.76 (S, 1H, ArH), 3.97~3.87 (m, 9H, CH_3), 3.83~3.78 (m, 3H, CH_3), ESI-MS: m/z 340.12 (M^+). Anal.Calcd for $C_{19}H_{20}N_2O_4$: C, 67.05; H, 6.92; N, 8.23. Purity: 96%.

1-Ethyl-N-(3,4,5-trimethoxyphenyl)-1*H*-indole-3-carboxamide

yellow solid, yield 76%, m.p. 138~139˚C; ^{1}H NMR (DMSO-*d*6, 300 MHZ) δ: 9.04 (S, 1H, ArH), 8.29 (d, J = 4.50 HZ, 1H,ArH), 8.12~7.94 (m, 1H, ArH), 7.58~7.56 (m, 1H, ArH), 7.26~7.29 (m, 2H, ArH, CH), 7.22~7.19 (m, 1H, ArH), 6.76 (s, 1H, ArH), 4.32~4.26 (m, 2H, CH_2), 3.92~3.86 (m, 6H, CH_3), 3.78~3.72 (m, 3H, CH_3), 1.08 (t, J = 4.63 HZ, 3H,CH_3), ESI-MS: m/z 354.16 (M^+). Anal.Calcd for $C_{20}H_{22}N_2O_4$: C, 67.78; H, 6.26; N, 7.90. Purity: 96%.

1-Propyl-N-(3,4,5-trimethoxyphenyl)-1*H*-indole-3-carboxamide

yellow solid, yield 86%, m.p. 148~149˚C; ^{1}H NMR (DMSO-*d*6, 300 MHZ) δ: 9.02 (S, 1H, ArH), 8.29 (d, J = 5.50 HZ,2H, ArH), 7.59~7.54 (m, 1H, ArH), 7.25~7.18 (m, 2H, ArH, CH), 6.34~6.22 (m, 2H, ArH), 4.26~4.22 (m, 2H, CH_2), 3.92~3.84 (m, 9H, CH_3), 1.89~1.80 (m, 2H, CH_2), 0.87 (t, J = 4.60 HZ, 3H,CH_3), ESI-MS: m/z 398.26 (M^+). Anal.Calcd for $C_{23}H_{30}N_2O_4$: C, 69.38; H, 7.56; N, 7.00. Purity: 95%.

1-Benzyl-N-(3,4,5-trimethoxyphenyl)-1*H*-indole-3-carboxamide

yellow solid, yield 69%, m.p. 144~146˚C; ^{1}H NMR (DMSO-d_6, 300 MHZ) δ: 8.92 (d, J = 5.66 HZ,1H, ArH), 8.26 (s, 1H, ArH), 7.59~7.54 (m, 1H, ArH), 7.69~7.48 (m, 2H, ArH, CH), 7.46~7.16 (m, 7H, ArH), 6.39~6.31 (m, 2H, ArH), 5.59~5.52 (m, 2H, CH_2), 3.92~3.78 (m, 9H, CH_3), ESI-MS: m/z 446.21 (M^+). Anal.Calcd for $C_{27}H_{30}N_2O_4$: C, 72.62; H, 6.76; N, 6.27. Purity: 98%.

^{1}H-NMR-compound **4**

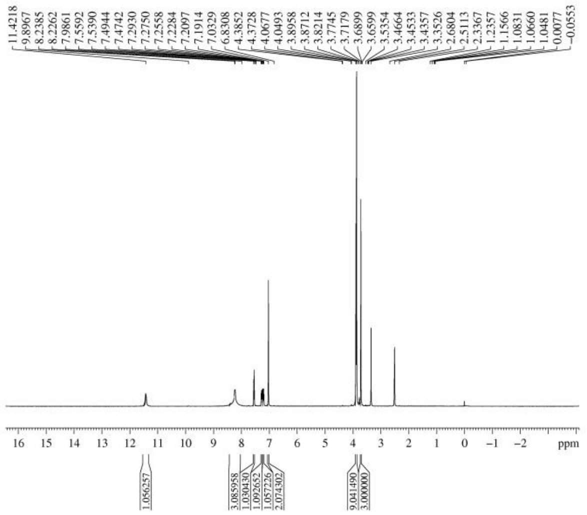

^{1}H-NMR-compound **5**

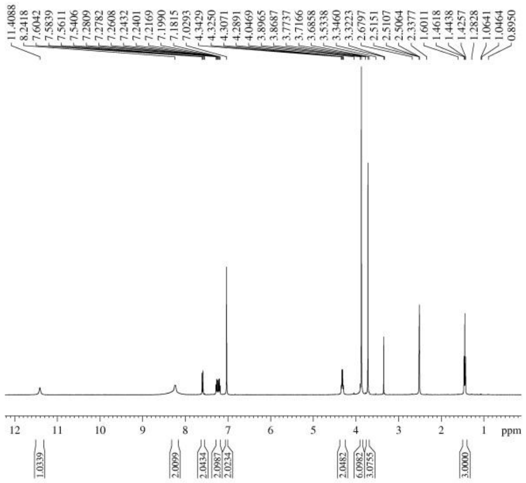

^{1}H-NMR-compound **6**

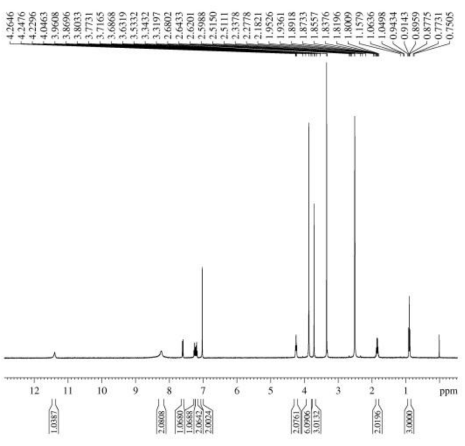

^{1}H-NMR-compound **7**

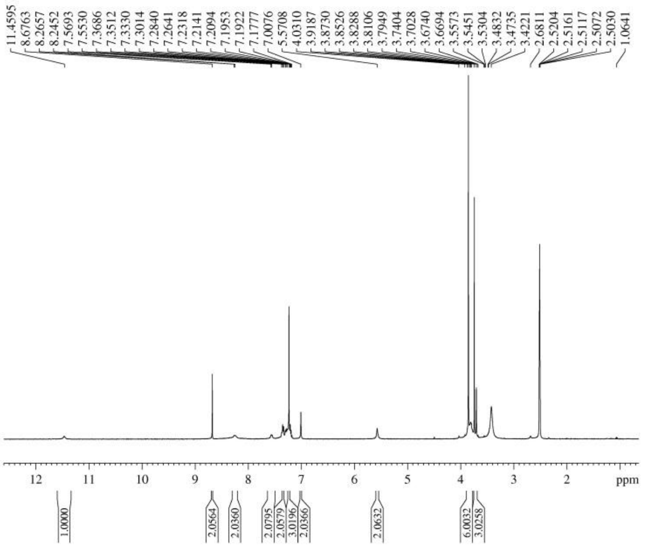

^{1}H-NMR-compound **10**

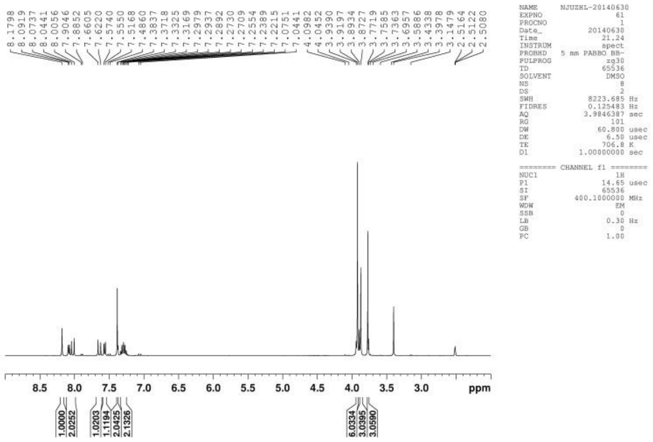

^{1}H-NMR-compound **11**

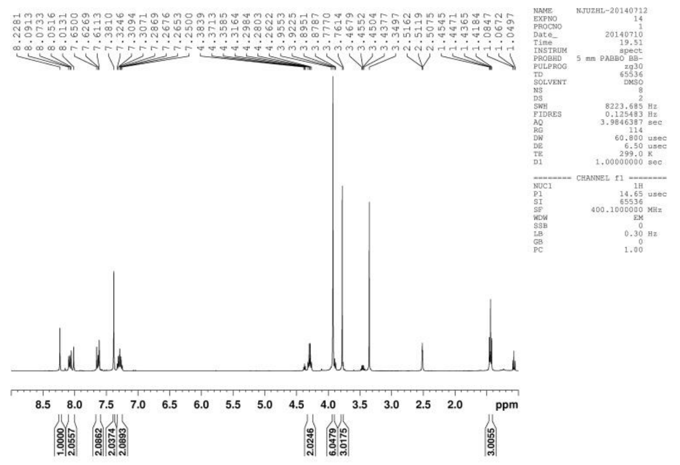

^{1}H-NMR-compound **12**

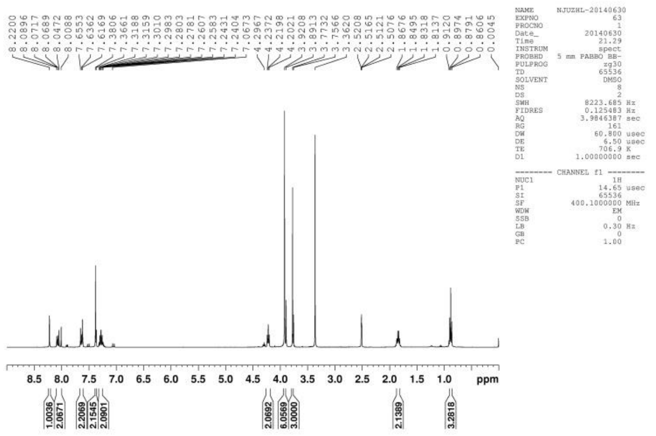

^{1}H-NMR-compound **13**

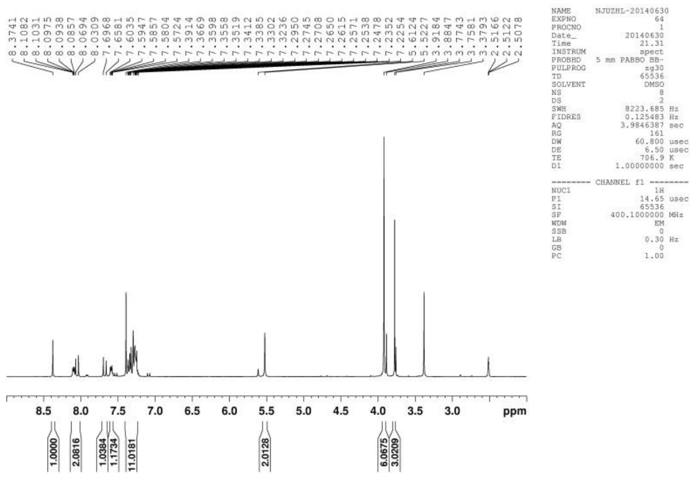

^{1}H-NMR-compound **16**

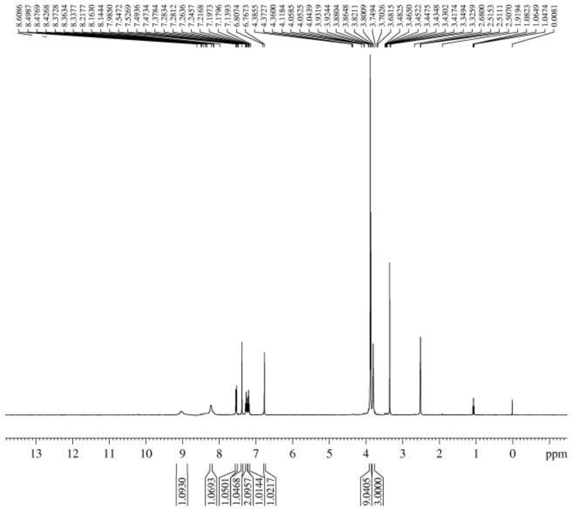

^{1}H-NMR-compound **17**

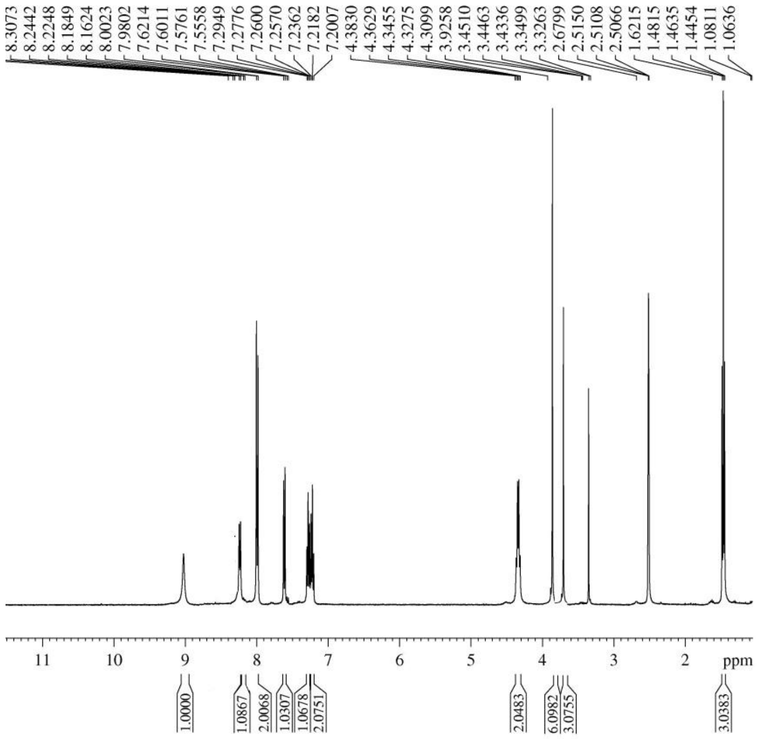

^{1}H-NMR-compound **18**

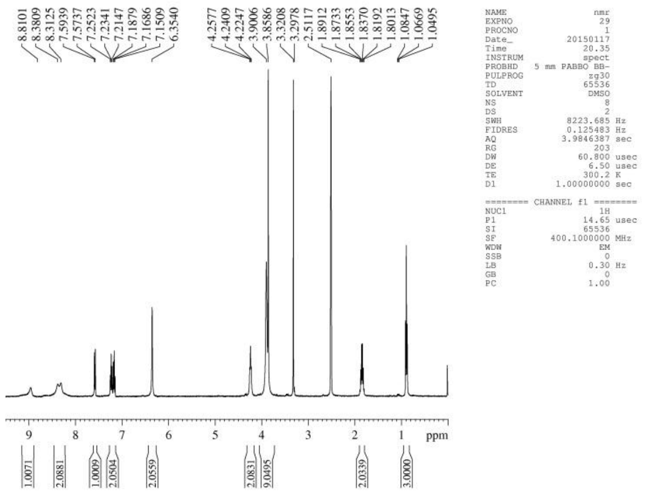

^{1}H-NMR-compound **19**

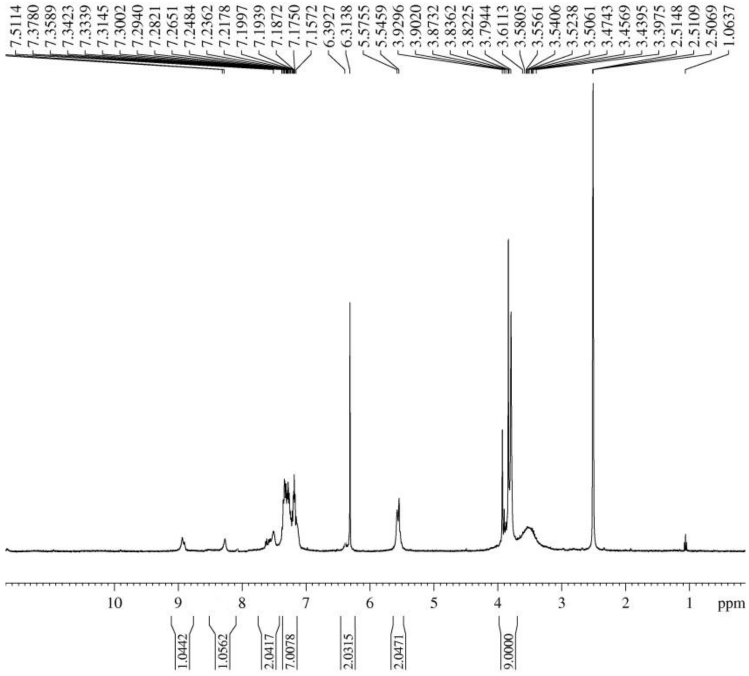

Supplement: Supplementary Information [file srep25387-s1.pdf]
